# Women and the War

**Published:** 1917-05

**Authors:** 


### Women and the War.

That the women of America are willing to bear their part of the burdens of war is not doubted by those who know the American women. As soon as it was known that a state of war existed, thousands of young women throughout the land began to prepare for Red Cross service, and today practically every college in the country is giving instruction in this class of military service to large classes of young women, who are anxious to go to the front if only they can be of real help to their countrymen or their allies.

Knowing that all of them cannot be used as nurses, they are preparing themselves by a study of food conservation and preservation in order that they may be of help in supplying the soldiers and civilians of this and other countries with the food with which to carry on the war, in most cases these two studies being carried on together in order that the students may fill either position to which they may be assigned. It is now planned that thousands of young college women shall go out through the country during the summer months teaching the women of the rural districts how to take care of the food crops produced on the farm.

It looks like the women are determined to demonstrate that from the beginning every woman will prove as valuable in winning the war as a man can possibly be on the battlefield. Such a demonstration is going to be worth much to American womanhood, for it will bring women to a realization of their usefulness in a way that will redound to their good for generations after the war.

At the New York Medical College and Hospital for Women, fifty young women medical students have offered their services to the United States in whatever way those services can be used. It is probable that practically every woman in the country who is practicing or studying medicine will make the same offer. Can the men do more?

This is in no sense a political journal, and it is not given to political prophecies, but it is so clear that all can now see that one result of this war, so far as America is concerned, will be that women will so demonstrate their loyalty and patriotism, as well as their intelligent interest in public affairs, that the men will gladly grant them the right of franchise.

